# Genetic consequences of climate change for northern plants

**DOI:** 10.1098/rspb.2011.2363

**Published:** 2012-01-04

**Authors:** Inger Greve Alsos, Dorothee Ehrich, Wilfried Thuiller, Pernille Bronken Eidesen, Andreas Tribsch, Peter Schönswetter, Claire Lagaye, Pierre Taberlet, Christian Brochmann

**Affiliations:** 1 Tromsø University Museum, 9037 Tromsø, Norway; 2 The University Centre in Svalbard, PO Box 156, 9171 Longyearbyen, Norway; 3 National Centre for Biosystematics, Natural History Museum, University of Oslo, PO Box 1172 Blindern, 0318 Oslo, Norway; 4 Department of Arctic and Marine Biology, Faculty of Biosciences, Fisheries and Economics, University of Tromsø, 9037 Tromsø, Norway; 5 Laboratoire d'Ecologie Alpine, CNRS UMR 5553, Université Joseph Fourier, PO Box 53, 38041 Grenoble Cedex 09, France; 6 Department of Organismic Biology, University of Salzburg, Hellbrunnerstrasse 34, 5020 Salzburg, Austria; 7 Botanical Institute, University of Innsbruck, Innrain 52, 6020 Innsbruck, Austria

**Keywords:** conservation genetics, *F*_ST_, genetic diversity, range reduction, species distribution model, species traits

## Abstract

Climate change will lead to loss of range for many species, and thus to loss of genetic diversity crucial for their long-term persistence. We analysed range-wide genetic diversity (amplified fragment length polymorphisms) in 9581 samples from 1200 populations of 27 northern plant species, to assess genetic consequences of range reduction and potential association with species traits. We used species distribution modelling (SDM, eight techniques, two global circulation models and two emission scenarios) to predict loss of range and genetic diversity by 2080. Loss of genetic diversity varied considerably among species, and this variation could be explained by dispersal adaptation (up to 57%) and by genetic differentiation among populations (*F*_ST_; up to 61%). Herbs lacking adaptations for long-distance dispersal were estimated to lose genetic diversity at higher rate than dwarf shrubs adapted to long-distance dispersal. The expected range reduction in these 27 northern species was larger than reported for temperate plants, and all were predicted to lose genetic diversity according to at least one scenario. SDM combined with *F*_ST_ estimates and/or with species trait information thus allows the prediction of species' vulnerability to climate change, aiding rational prioritization of conservation efforts.

## 1. Introduction

When addressing impacts of climate change on biological diversity, most studies treat a species as a unit and thus ignore intraspecific genetic variation [1,2]. Maintaining genetic diversity within a species is crucial for its ability to adapt both in the short-term and long-term survival [3–5]. Species may respond to climate change by local adaptation [6,7], range shift [8,9], range reduction [10] or a combination of these [6]. While range shift may alter the genetic diversity within species [11,12], range reduction is most likely to cause loss of genetic diversity [13,14] and may therefore severely limit the species' ability to adapt to a changing climate [4].

Most species adapted to cold environments are expected to suffer range reduction following climate warming [7,10]. In animals, demographically challenged populations showed 22–26% reduction in genetic diversity relative to healthy populations [15], and two of three studied lizard species and one cold-adapted aquatic mayfly were expected to experience genetic depauperation owing to climate change [16,17]. By contrast, in the only plant species studied to date, the wind-dispersed dwarf shrub *Salix herbacea*, a loss of 50 per cent of its European range was estimated to cause a loss of only 5 per cent of its genetic diversity because of its high dispersal ability and history of broad-fronted postglacial colonization [18]. Thus, species traits that influence the distribution of genetic diversity within and among populations [19,20] may also determine the susceptibility of species to genetic diversity loss when their ranges are reduced, and their consequent vulnerability to loss of evolutionary potential [3].

Here, we use published and new amplified fragment length polymorphism (AFLP) data combined with a randomization procedure to estimate loss of genetic diversity under increasing loss of range for 27 northern plant species, and explore the effect of different species traits. We also explore whether genetic differentiation among populations (*F*_ST_), which is available for thousands of species, can be used as a proxy for predicting the genetic vulnerability of species to climate change. Finally, we use an ensemble of species distribution models (SDMs) to estimate the expected range reduction and associated loss of genetic diversity under two different global circulation models (GCMs) and two emission scenarios. Our results show that the expected genetic consequences of climate change differ markedly among species according to their adaptations to seed dispersal and growth forms, and that it is possible to predict the genetic consequences of range reduction by combining species modelling approaches with prior knowledge on species traits and/or *F*_ST_ estimates.

## 2. Material and methods

### (a) Species

We analysed 27 plant species typically occurring in the bioclimatic zones at the tree line and beyond, i.e. the alpine and arctic zones. Information on the following species traits was compiled from the literature and databases: dispersal adaptation, growth form, pollination mode, breeding system, northernmost bioclimatic zone where it occurs, temperature tolerance and current geographical distribution type (see the electronic supplementary material).

### (b) Loss of genetic diversity

We analysed 73–958 individual plants from 14 to 131 local populations of each species and assessed levels of genetic diversity from AFLP data (78–334 markers per species, see the electronic supplementary material). For 24 of the species, full details of data collection and genetic structuring have been published elsewhere (electronic supplementary material, table S1).

**Figure 1.**
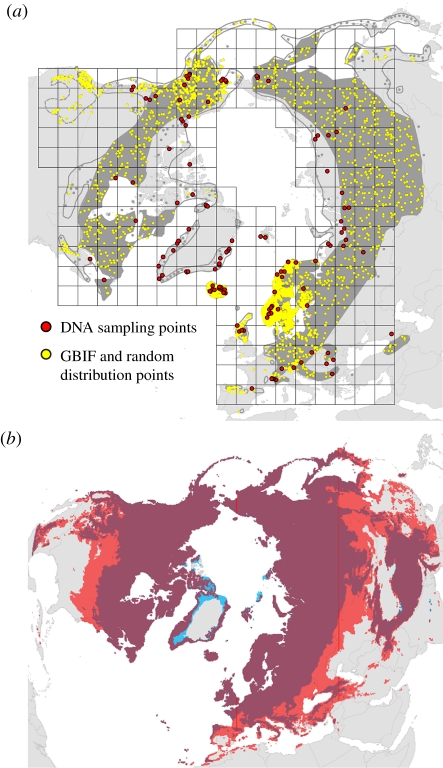
Estimating loss of genetic diversity and range reduction, exemplified by data for *Vaccinium uliginosum*. (*a*) DNA sampling points, the 500 × 500 km grid overlaying the sampling points to adjust for variation in sampling intensity, and distribution sampling points used for modelling present and future distribution. GBIF, Global Biodiversity Information Facility. Dark grey shows current distribution. (*b*) Potential present and future (year 2080) distribution habitats overlaid to show lost (red), stable (purple) and future new habitat (blue). In this example, a 26% range reduction was estimated for the A2 emission scenario and CCM3 global circulation model, and there were 53 grid cells of 500 × 500 km that contained samples. The predicted loss of 26% of the range corresponded to a loss of 14 grid cells.

To estimate loss of genetic diversity expected as a consequence of range loss, we first divided the total study area according to an arbitrary grid of 500 × 500 km cells using ArcMap v. 9.2 and the Lambert azimuthal-equal area projection of the Northern Hemisphere (figure 1). This was performed to account for differences in sampling intensity in different areas. For each species, only grid cells containing sampling localities were retained. The genetic consequences of range reduction were estimated as the loss of AFLP markers occurring from randomly removing an increasing number of grid cells. This procedure was repeated 1000 times to create a look-up table for minimum, maximum, mean and median number of markers lost for increasing numbers of grid cells removed.

We expressed the number of AFLP markers lost relative to genetic diversity among individuals. Considering that a species would have lost all its genetic diversity when the last remaining population consists of identical individuals, we defined total loss of diversity as the total number of markers minus average number of markers per individual. The estimated loss of genetic diversity (*G*_loss_) was thus expressed relative to this total loss as: *G*_loss_ = average number of markers lost/(total number of markers − average number of markers per individual). We investigated the sensitivity of our estimates to variation in grid sizes and to geographical patterns in loss of genetic diversity (loss of adjacent grid cells after starting at a random point, see the electronic supplementary material).

### (c) Range reduction and range gain

To estimate range reduction and gain, we used SDMs relating observed species occurrences to environmental variables [21]. These models are reported to be of moderate to good quality with respect to reconstructing recent and Holocene past distributions [22,23]. We selected three climatic variables regarded as important in determining plant distributions [24]: annual sum of precipitation, mean maximum temperature of the warmest month and mean minimum temperature of the coldest month. These variables for current (1961–1990) and future (2071–2100) conditions were extracted from the Climate Research Unit (http://www.cru.uea.ac.uk/) and the Worldclim (http://www.worldclim.org/) data centre at a 10′ resolution. Future climates were represented by Community Climate Model version 3 (CCM3) and Hadley Centre Coupled Model version 3 (HadCM3) GCMs for A2 (+3.2°C) and B2 (+1.0°C) emission scenarios [25].

Observed distributions were digitized at the circumboreal/circumarctic scale from Hultén & Fries' [26] distribution maps. Because these polygon maps represented the extent of occurrence of the species, we selected a number of random presences inside these polygons (resulting in one point for 1000 km^2^). We also added validated occurrence points from the Global Biodiversity Information Facility (http://www.gbif.org/), and from our own field sampling locations (electronic supplementary material, table S1). Selected climatic variables were extracted for these points. We created spatially random pseudo-absences (same number as presences) outside of these climatic boundaries [27]. All presence and absence locations were summarized in a 7.5 km resolution grid (North Pole Lambert azimuthal-equal area projection, 1.4 million cells) to decrease the pervasive effects of spatial autocorrelation. Current and future climate were then resampled in the same 7.5 km grid to run the projections.

SDMs were calibrated within the *biomod* package in R [28], which capitalizes on seven widely used techniques (generalized linear models, generalized additive models, classification tree analysis, boosted regression trees, random forest, multiple adaptive regression splines and mixture discriminant analysis) to provide an ensemble of spatial projections. Model calibration was performed on a random sample of the data (70%) and model evaluation was carried out on the remaining 30 per cent with the true skill statistic (TSS) [29] and the area under the receiver-operating characteristic plot [30]. Calibrated models were then used to project current and future suitable climatic habitats over the Northern Hemisphere north of 20°N latitude. We transformed the current occurrence probabilities into presences/absences using two methods; the thresholds that maximize (i) both the percentage of presences and absences correctly predicted, and (ii) the TSS. We used the same thresholds to convert future projections. To investigate the uncertainty coming from the overall modelling approach, we used the projections from the seven different SDMs, transformed into presence–absence using the two above-mentioned approaches (7 × 2 = 14 projections for current climate and each pair global circulation × emission scenario) to estimate percentage of range reduction, range gain and range change.

Because the above-mentioned models can be overly complex, we also performed a simple rectilinear envelop model (surface range envelop [31]). The envelope is defined by identifying maximum and minimum values for each variable from observed presence of a species. Any presence with all variables falling between these maximum and minimum limits was included within the range that depicts the climatic conditions within which the species have been recorded. Being less constrained, the potential range is usually larger than those estimated by more complex models.

### (d) Loss of genetic diversity in the year 2080

The proportion of genetic diversity likely to be lost in the year 2080 was determined by evaluating the number of grid cells corresponding to the estimated range reduction (see figure 1), and then using the look-up table described earlier to determine the expected loss of genetic diversity for this value. By choosing to use only predicted range loss, we ignore that the levels of genetic diversity in the new range may increase owing to increased mutation rate or introgression [32]. We considered this to be suitable given that genetic diversity in a new range has been shown to typically represent a subset of the genetic diversity in the source populations owing to bottlenecks and founder events [11,12,32], and given that we are considering a short time span. Based on a similar argument, we also ignore that genetic diversity may be retained if genotypes in extirpating populations manage to escape into the stable parts of the range; studies have shown that populations from southern portions of a geographical range may contribute little to genetic diversity of more northern populations [33–35].

### (e) Statistical analyses

For each species, genetic differentiation among all sampled populations (*F*_ST_) was estimated by an analysis of molecular variance using the software Arlequin v. 3.0 [36].

We tested for correlations among species' traits using linear models for correlations involving the continuous variables like northernmost bioclimatic zone and temperature, and *χ*^2^ tests for 2 ×2 contingency tables to test the strength of association between categorical variables. We found a significant correlation between growth form and dispersal adaptation (electronic supplementary material, table S2). As dispersal has higher impact on genetic diversity than growth form [19,20], we kept dispersal adaptation. There was also a significant correlation between northernmost bioclimatic zones and both dispersal adaptations and breeding system (electronic supplementary material, table S2). As the thermal tolerance of the species was taken into account through maximum and minimum temperatures, we omitted the northernmost bioclimatic zone from further analyses. There was a marginal significant interaction between breeding system and distribution, but this was ignored.

Expected loss of genetic diversity, which was expressed as proportion, was arcsine (square root) transformed in all analyses, which according to Shapiro–Wilk tests normalized distribution in all cases (*p* > 0.05) except for at 10 per cent range reduction.

Linear models were used: (i) to investigate the effect of dispersal adaptations, pollination mode, breeding system, temperature tolerance and current geographical distribution type on loss of genetic diversity given a certain range loss, (ii) the usefulness of genetic differentiation (*F*_ST_) as a predictor for the rate of genetic loss, and (iii) the effect of range reduction by 2080 on loss of genetic diversity. Model selection was performed by Akaike's Information Criterion corrected (AICc) for small sample size ([37]; electronic supplementary material, table S3). This model selection criterion balances model complexity and model fit. The *Δ*AICc of the best-fit model is zero, and models with *Δ*AICc ≤ 2 have some support [37]. Candidate models included models with single predictors and with all different combinations of two predictors (electronic supplementary material, table S3). The residuals of all selected models were checked using diagnostic plots to see if they satisfactorily met the assumptions of linear models (stable variance). Some outliers were discovered, but the same or stronger correlations were found when we excluded the outliers. To check for possible bias owing to unequal numbers of populations, individuals per populations or number of AFLP markers, each of these variables was added to the best candidate model as additional explanation variables. All analyses mentioned above were performed in R v. 2.12 [38].

A possible problem with the above-mentioned analyses is that species are treated as statistically independent units. This approach compares the extant species situated at the tip of the phylogenies (TIP). As plant traits are likely to be correlated for phylogenetically related species, analyses taking into account phylogeny may reveal different results. Therefore, we also tested the effect of traits using phylogenetic-independent contrasts (PICs) [39]. We constructed a phylogenetic tree (electronic supplementary material, figure S2) using the online software Phylomatic (http://www.phylodiversity.net/phylomatic/phylomatic.html) and choosing the angiosperm consensus tree [40]. A polytomy in Ericaceae was resolved according to Kron *et al*. [41]. The gymnosperm *Juniperus communis* was regarded as sister to all angiosperms. All branch lengths were assigned a value of 1. The categorical variables such as dispersal, range, breeding, pollination and life form were coded as dummy variables (0, 1). The PIC analyses were run in Compare v. 4.6b [42]. A rough 95% CI for the regression slopes was estimated as ±1.96 s.e. Effects were considered significant, if the CI excluded zero.

## 3. Results

**Figure 2.**
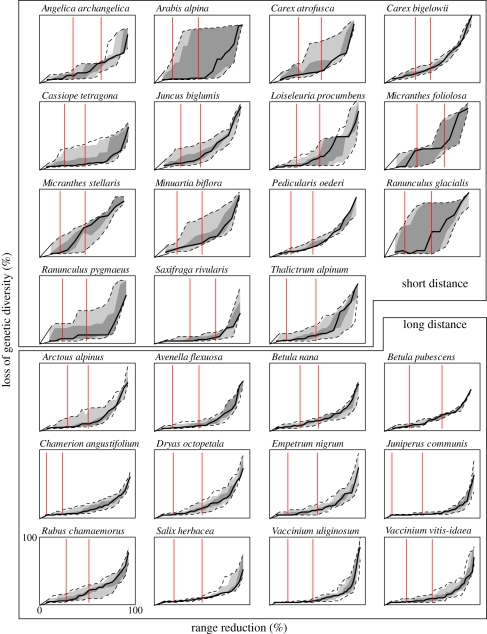
Estimated loss of genetic diversity as a function of decreasing range for 27 northern plant species. The bold line refers to the median; the dark grey shaded area refers to 50% CI; the light grey shaded area refers to 90% CI; and the dashed lines refer to minimum and maximum loss of genetic diversity. Vertical red lines show minimum and maximum range reduction expected by the year 2080 by any of seven species distribution models, two emission scenarios and two global circulation models (see §2).

In all cases, the relationship between loss of genetic diversity and loss of range was nonlinear, indicating that the majority of genetic markers are shared among geographical regions (figure 2). The rather narrow 90% CI observed for the majority of species indicate that loss of genetic diversity was in most cases rather independent on the order in which parts of the range were lost, and that loss could be estimated with high precision. For some species, however, uncertainties were high, especially *Arabis alpina* and *Ranculus glacialis* (figure 2). The median, minimum and maximum loss of genetic diversity remained largely similar in the analysis modelling loss of geographically adjacent areas, but the CI increased (see the electronic supplementary material, figure S1).

Loss of genetic diversity was best explained by dispersal adaptation alone, or by dispersal adaptation combined with minimum temperature of the coldest month, or with total distribution range according to the AICc (electronic supplementary material, table S3). These results were robust to differences in number of populations, mean number of individuals per population and total number of polymorphic markers (see the electronic supplementary material). Our estimates indicated that species without adaptations to long-distance dispersal (and/or herbaceous species) will lose genetic diversity at about twice the rate of species adapted to long-distance dispersal by animals or wind (and/or woody species; figure 2 and table 1). The results were similar for TIP and PIC analyses (table 1). However, dispersal adaptation was strongly correlated with growth form in our set of species (table 2 and electronic supplementary material, S2); thus, the effect of these two traits could not be distinguished. Importantly, but not unexpectedly, the rate of genetic loss with range reduction was estimated to be higher for species with higher genetic differentiation among populations (*F*_ST_; table 1). Most of the species lacking adaptation to dispersal had *F*_ST_ values greater than 0.5, whereas all but one species adapted to long-distance dispersal had *F*_ST_ values less than 0.5 (table 2).

**Table 1.** Parameter estimates and 95% CI (in parentheses) for the models explaining arcsine (square root) transformed loss of genetic diversity (*G*_loss_) for 10–90% range reduction using conventional regression (TIP). (For comparison, results of phylogenetic-independent contrast analyses (PICs) are also shown. Parameters are the adaptations for dispersal (short- or long-distance) and genetic differentiation among populations (*F*_ST_). The total *r*^2^ and significance level (*p*) is given for each model.)

range reduction (%)	*G*_loss_ ∼ dispersal				*G*_loss_ ∼ *F*_ST_			
	intercept	dispersal = short	*r*^2^	*p*-value	intercept	*F*_ST_	*r*^2^	*p*-value
TIP								
10	0.05 (0.02–0.09)	0.05 (0, 0.10)	0.14	0.06	0.04 (−0.02–0.10)	0.07 (−0.03–0.18)	0.07	0.17
20	0.11 (0.07–0.15)	0.08 (0.03–0.14)	0.28	0.01	0.07 (0.01 0.13)	0.17 (0.06–0.28)	0.29	0
30	0.14 (0.08–0.20)	0.15 (0.07–0.23)	0.35	0	0.05 (−0.04–0.14)	0.34 (0.18–0.50)	0.44	0
40	0.19 (0.13–0.25)	0.17 (0.09–0.25)	0.43	0	0.10 (0.01–0.19)	0.37 (0.20–0.53)	0.46	0
50	0.25 (0.19–0.31)	0.20 (0.12–0.28)	0.52	0	0.16 (0.07–0.26)	0.39 (0.22–0.56)	0.48	0
60	0.29 (0.23–0.35)	0.24 (0.15–0.32)	0.56	0	0.19 (0.09–0.30)	0.45 (0.26–0.65)	0.48	0
70	0.36 (0.29–0.44)	0.26 (0.17–0.36)	0.56	0	0.25 (0.13–0.37)	0.51 (0.30–0.73)	0.49	0
80	0.46 (0.36–0.55)	0.34 (0.21–0.47)	0.53	0	0.30 (0.14–0.45)	0.69 (0.42–0.97)	0.52	0
90	0.64 (0.51–0.76)	0.46 (0.30–0.62)	0.57	0	0.40 (0.22–0.58)	0.98 (0.66–1.30)	0.61	0
PIC								
10	0.01 (−0.03, −0.01)	−0.01 (−0.01, −0.01)	0.31	<0.05	0 (−0.04, 0.04)	0.02 (0, 0.04)	0.18	>0.05
20	0.04 (−0.02, −0.02)	−0.03 (−0.05, −0.03)	0.39	<0.05	0 (−0.06, 0.06)	0.05 (0.03, 0.07)	0.36	<0.05
30	0.09 (−0.05, −0.05)	−0.07 (−0.11, −0.07)	0.30	<0.05	−0.02 (−0.12, 0.08)	0.17 (0.11, 0.23)	0.62	<0.05
40	0.12 (−0.06, −0.06)	−0.09 (−0.13, −0.09)	0.33	<0.05	−0.01 (−0.17, 0.15)	0.19 (0.11, 0.27)	0.52	<0.05
50	0.16 (−0.06, −0.09)	−0.13 (−0.19, −0.13)	0.42	<0.05	−0.02 (−0.22, 0.18)	0.24 (0.14, 0.34)	0.49	<0.05
60	0.21 (−0.04, −0.10)	−0.16 (−0.24, −0.16)	0.43	<0.05	0 (−0.27, 0.27)	0.28 (0.14, 0.42)	0.42	<0.05
70	0.29 (−0.04, −0.12)	−0.21 (−0.31, −0.21)	0.44	<0.05	0.02 (−0.31, 0.35)	0.36 (0.20, 0.52)	0.43	<0.05
80	0.39 (−0.08, −0.14)	−0.27 (−0.41, −0.27)	0.39	<0.05	0.01 (−0.46, 0.48)	0.51 (0.29, 0.73)	0.45	<0.05
90	0.58 (0.13, −0.05)	−0.32 (−0.44, −0.32)	0.49	<0.05	0.13 (−0.26, 0.52)	0.62 (0.42, 0.82)	0.61	<0.05

**Table 2.** Estimated range reduction (*R*_red._,%, median of seven techniques) and associated estimated loss of genetic diversity (*G*_loss_,%) for 27 northern plant species, according to the A2 and B2 emission scenarios and the CCM3 and HadCM3 global circulation models. (Dispersal: S, short-distance; L, long-distance. Growth form: H, herbaceous; W, woody. Genetic differentiation among populations (*F*_ST_).)

species	dispersal	growth form	*F*_ST_	A2 CCM3		A2 HadCM3		B2 CCM3		B2 HadCM3	
				*R*_red._	*G*_loss_	*R*_red._	*G*_loss_	*R*_red._	*G*_loss_	*R*_red._	*G*_loss_
*Angelica archangelica*	S	H	0.40	51	15	63	22	38	9	51	15
*Arabis alpina*	S	H	0.86	24	8	34	15	17	6	26	8
*Arctous alpinus*	L	W	0.32	41	6	49	9	28	3	36	5
*Avenella flexuosa*	L	H	0.24	25	1	33	2	18	1	26	1
*Betula nana*	L	W	0.20	45	7	49	8	35	5	39	6
*Betula pubescens*	L	W	0.05	38	6	55	11	29	4	41	6
*Carex atrofusca*	S	H	0.93	47	23	49	23	35	14	39	18
*Carex bigelowii*	S	H	0.44	45	19	47	19	34	11	38	13
*Cassiope tetragona*	S	W	0.29	43	9	47	10	32	5	37	7
*Chamerion angustifolium*	L	H	0.24	8	0	11	1	6	0	6	0
*Dryas octopetala*	L	W	0.46	26	3	34	4	18	2	25	3
*Empetrum nigrum* s.lat.	L	W	0.56	29	2	39	4	20	2	27	2
*Juncus biglumis*	S	H	0.85	44	23	45	23	33	15	36	19
*Juniperus communis*	L	W	0.27	11	0	17	0	7	0	10	0
*Loiseleuria procumbens*	S	W	0.68	41	4	51	6	29	2	39	4
*Micranthes foliolosa* s.lat.	S	H	0.66	60	30	48	24	43	20	37	20
*Micranthes stellaris*	S	H	0.68	30	8	43	19	22	3	33	11
*Minuartia biflora*	S	H	0.92	44	21	48	27	34	16	39	21
*Pedicularis oederi*	S	H	0.55	31	6	42	11	22	5	29	6
*Ranunculus glacialis*	S	H	0.60	28	8	41	17	21	8	32	12
*Ranunculus pygmaeus*	S	H	0.94	41	21	49	24	32	21	40	21
*Rubus chamaemorus*	L	H	0.40	43	9	50	11	31	6	38	7
*Salix herbacea*	L	W	0.40	28	3	39	4	22	2	31	3
*Saxifraga rivularis*	S	H	0.58	61	9	55	5	46	2	42	2
*Thalictrum alpinum*	S	H	0.34	23	2	30	4	16	1	22	2
*Vaccinium uliginosum*	L	W	0.35	26	0	34	0	19	0	25	0
*Vaccinium vitis-idaea*	L	W	0.38	37	3	47	4	24	1	33	3
average for all species				36	9	43	11	26	6	32	8

The prediction power of the SDMs was generally high (area under the curve > 0.98, TSS > 0.85, electronic supplementary material, table S4). For 18 of the species, the predicted range reduction (median based on seven techniques) exceeded 40 per cent for at least one emission scenario and circulation model (table 2). Range reduction was on average higher under emission scenario A2 (‘business as usual’, 36–43% reduction) than under B2 (‘reduced CO_2_ emission’, 26–32% reduction), as expected as the A2 scenario anticipates a more severe climate change than the B2 scenario (table 2). The range gain was generally considerably lower than the range reduction, and on average the range change was −24 per cent for A2 CCM3, −30 per cent for A2 HadCM3, −16 per cent for B2 CCM3 and −22 per cent for B2 HadCM3 (electronic supplementary material, table S5).

According to the median of seven techniques, all species except two very widespread, abundant and bird-dispersed ones (*J. communis* and *Vaccinium uliginosum*) were predicted to lose some of their present genetic diversity by 2080 (table 2). The estimated loss of genetic diversity in *Micranthes foliolosa* was 30 per cent, and six species were estimated to lose more than 20 per cent of their genetic diversity according to at least one scenario (table 2). However, there was a large gap between minimum and maximum estimated range reduction, and all species were expected to lose range under some models (electronic supplementary material, table S5). In the worst case scenario, assuming that the model estimating the maximum range reduction will be realized, and that the corresponding loss of genetic diversity will be at the maximum value (crossing point between right red bar and upper dashed line in figure 2), all species were expected to lose some genetic diversity, one-third of them greater than 50 per cent.

The median estimated loss of genetic diversity differed only slightly among the emission scenarios and GCMs (table 2). Species expected to lose more of their range were also prone to more severe genetic loss: range reduction alone explained 66–74% of the variation in estimated loss of genetic diversity among species (not shown).

## 4. Discussion

Our results show that species traits which are well known to influence patterns of genetic diversity within and among populations, also affect the predicted loss of genetic diversity, and thereby the susceptibility of species to genetic depauperation under range reduction. As our modelling predicts range reduction and loss of genetic diversity by 2080 in all 27 species according to at least one scenario, it can be expected that climate warming will have a major impact on the future range sizes and levels of genetic diversity in northern plant species. Because genetic diversity is important not only for species' persistence and evolutionary potential [3,4], but also for community structure and ecosystem resilience [43], climate change-induced loss of genetic diversity may be expected to affect all levels of biodiversity.

### (a) Vulnerability to loss of genetic diversity

The precision of our predicted loss of genetic diversity largely depended on the geographical distribution of genetic diversity within the species. For species with a rather even level of genetic diversity throughout most of its distribution range, as e.g. in *Carex bigelowii* and *S. herbacea* [18,44,45], the prediction intervals are fairly small with the minimum and maximum predicted loss of genetic diversity close to the median, indicating that the effect of range reduction is rather independent on which part of the range is lost (figure 2). However, for species such as those with high levels of genetic diversity in southern alpine areas and hardly any diversity in northern areas, as observed in *A. alpina* and *R. glacialis* [46,47], the future loss of genetic diversity will strongly depend on which part of the range is lost. As southern populations are most likely to get lost (cf. figure 1), the expected loss of genetic diversity is likely to be at the maximum estimates (figure 2), and thus cause severe genetic depauperation.

Dispersal adaptations appear to be important in determining the rate of loss of genetic diversity. This is not surprising, as dispersal adaptations influence the level of genetic differentiation among plant populations [19,20,48]. Also among our species, those adapted to long-distance dispersal had lower *F*_ST_ values than the species lacking such adaptations. However, given the strong correlation between dispersal adaptations and growth form in our dataset, the differences we observed among short- and long-distance dispersers may be based on a combination of these two species traits. Growth form may indeed also contribute to shaping genetic patterns, as woody species (especially trees) often have a larger stature and lower population densities, which could result in higher pollen and seed dispersal [19]. A recent meta-analysis showed, however, that woody and herbaceous species may experience similar loss of genetic diversity, a consequence of habitat fragmentation [14]. Woody species may also suffer more from inbreeding depression than herbaceous plants, and thus have a higher selection pressure than inbreds [49]. In addition, many species among our long-distance dispersers occur abundantly as vegetation dominants with very high seed outputs, which may further facilitate long-distant gene flow. The typically high levels of gene flow among populations of long-distance-dispersed woody species [19,20] create a genetic pattern which make species less vulnerable to loss of genetic diversity during range reduction. As loss of genetic diversity is expected to adversely affect the ability of populations to evolve and cope with environmental change and thereby increase the risk of extinction [50,51], short-distance-dispersed herbs may be expected to enter the extinction vortex more rapidly. Using knowledge on species traits may thus help us forecast which species are at risk.

The strong correlation we found between *F*_ST_ and loss of genetic diversity, suggests that *F*_ST_ values can be used as a proxy for predicting the genetic vulnerability of species to climate change. However, as estimates of *F*_ST_, *G*_ST_ or related measures of population differentiation depend on the type of genetic markers used, the geographical scale of the study, the number of individuals and populations sampled, and the estimator used [52–54], the actual *F*_ST_ estimates should be evaluated in the light of these sources of bias.

Our AFLP-based predicted loss mainly represents loss of neutral genetic diversity [55], whereas species' abilities to adapt and survive climate change mainly depend on adaptive genetic variation. Although methods for estimating range-wide levels of adaptive genetic variation are under development [56], it is still not feasible to apply them to approximately 10 000 samples of 27 different species as studied here. Neutral genetic variation can be expected to be lost at a rate similar to that of adaptive genetic variation [57], but the differentiation among populations is typically higher when based on adaptive than on neutral genetic diversity [58,59]. It is therefore likely that our estimates are underestimating the actual loss of evolutionary potential.

### (b) Prediction for 2080

Overall, the range changes modelled for these northern species in 2080 (electronic supplementary material, table S5) were slightly higher than those modelled for temperate tree species under the HadCM3 GCM and A2 (30% versus 22%) or B2 (22% versus 19%) emission scenarios [60], and that modelled for 84 Danish temperate species (29% and 12% for scenarios A2 and B2, respectively) [61]. In addition, alpine species are expected to lose more habitat than boreal species [10]. Thus, it is reasonable to suggest that susceptibility to climate warming-induced range reduction and associated loss of genetic diversity will be highest in species restricted to cold climates.

Currently, The International Union for Conservation of Nature [62] red list criteria do not take genetic diversity into account, but species with a population reduction expected in the future (up to a maximum of 100 years) should be listed as critically endangered, endangered or vulnerable if the expected range reduction is greater than or equal to 80 per cent, greater than or equal to 50 per cent, or greater than or equal to 30 per cent, respectively. As many as 26 of the 27 rather common and widely distributed species studied here fall within these categories according to at least one emission scenario, modelling technique and global distribution model (figure 2). Given the high number of species that are expected to become vulnerable or threatened by 2080 [10], it will become difficult to prioritize species for conservation. As our study indicates that the genetic consequences of range reductions are remarkably different among species, genetic parameters should be considered in future management assessment.

SDMs are widely used for management purposes [63]. The prediction power of these models in our dataset was high, and we show that they may also, combined with genetic data, be a useful tool for estimating expected loss of genetic diversity. Nevertheless, these models do not take into account mechanisms such as changes in biotic interactions, potential for rapid adaptations or time lag. Although some newly developed spatially explicit models are able to account for these factors [64,65], they are not ready to be implemented for a large set of species and to be run over the whole arctic–alpine region. When species shift ranges, some populations may persist at the rear edge and conserve local genetic variants. If this is the case, then our approach may overestimate habitat loss and associated loss of genetic diversity. Research on rear-edge populations is still limited [34], and their response to climate change may be challenging to model [2]. Populations may persist if they are able to colonize adjacent microhabitats in heterogeneous landscapes [2,34], if they are able to endure long periods without recruitment (owing to, e.g. clonal growth, persistent seed bank, long lifespan, etc.) [2,34], and/or because they adapt to the changing climate [7,66,67]. We assume that switching of microhabitats is equally likely in short- and long-distance-dispersed species. Extinction lags are more pronounced in long-lived woody species than in herbaceous species [9], but this might be outweighed by the faster evolutionary rate in herbaceous than woody species [68]. Also, as good dispersal ability is likely to enhance both the ability of range change and adaptive evolution [67], we think that any uncertainties in the models are likely to increase rather than decrease the differences we observed in susceptibility to loss of genetic diversity in woody, long-distance-dispersed compared with herbaceous, short-distance-dispersed species.

## 5. Conclusions

Our study shows that *F*_ST_ values, which are available for a wide range of species, as well as species traits such as dispersal adaptation and growth form, can be used to predict a species' susceptibility to loss of genetic diversity following climate change. As it is important to assess and commence management actions before genetic diversity is lost [69], we advocate to combine SDM with data on genetic differentiation and/or species traits to predict which species are at highest risk of losing genetic diversity in a changing climate. Such an approach will facilitate rational prioritization of conservation efforts.
